# Pyrrolidine Dithiocarbamate (PDTC) Inhibits DON-Induced Mitochondrial Dysfunction and Apoptosis via the NF-*κ*B/iNOS Pathway

**DOI:** 10.1155/2018/1324173

**Published:** 2018-11-25

**Authors:** Dan Wan, Qinghua Wu, Wei Qu, Gang Liu, Xu Wang

**Affiliations:** ^1^Hunan Provincial Key Laboratory of Animal Nutritional Physiology and Metabolic Process, Key Laboratory of Agro-Ecological Processes in Subtropical Region, Institute of Subtropical Agriculture, Chinese Academy of Sciences, National Engineering Laboratory for Pollution Control and Waste Utilization in Livestock and Poultry Production, Changsha, Hunan 410125, China; ^2^National Reference Laboratory of Veterinary Drug Residues (HZAU) and MAO Key Laboratory for Detection of Veterinary Drug Residues, Wuhan 430070, China; ^3^MOA Laboratory for Risk Assessment of Quality and Safety of Livestock and Poultry Products, Wuhan 430070, China; ^4^Hubei Collaborative Innovation Center for Animal Nutrition and Feed Safety, Wuhan 430070, China; ^5^College of Life Science, Yangtze University, Jingzhou, China; ^6^Department of Chemistry, Faculty of Science, University of Hradec Kralove, Hradec Kralove, Czech Republic

## Abstract

Oxidative stress is closely linked to the toxic responses of various cell types in normal and pathophysiological conditions. Deoxynivalenol (DON), an inducer of stress responses in the ribosome and the endoplasmic reticulum (ER), causes mitochondrial dysfunction and mitochondria-dependent apoptosis through oxidative stress in humans and animals. The NF-*κ*B pathway, which is closely linked to oxidative stress, is hypothesized to be a critical signaling pathway for DON-induced toxicity and is a potential target for intervention. The present study was conducted to explore the protective effects of pyrrolidine dithiocarbamate (PDTC) from the toxic effects of DON in rat anterior pituitary GH3 cells. Our results showed that DON activated the NF-*κ*B transcription factors and induced cellular oxidative stress, mitochondrial dysfunction, and apoptosis. Morphological studies using transmission electron microscopy (TEM) and cell apoptosis analyses suggested that PDTC prevented DON-induced mitochondrial dysfunction and apoptosis, probably by preventing the DON-induced translocation of NF-*κ*B p65 into the nucleus, and by inhibiting DON-induced iNOS expression. This led to the blocking of the NF-*κ*B pathway and inhibition of iNOS activity.

## 1. Introduction

Oxidative stress is closely linked to toxic responses of various cell types in normal and pathophysiological conditions. Deoxynivalenol (DON), produced by the *Fusarium graminearum* and *F. culmorum* species, is an inducer of stress responses in the ribosome and the endoplasmic reticulum (ER). It causes mitochondrial dysfunction and mitochondria-dependent apoptosis through oxidative stress [1, 2]. The consumption of DON-contaminated products causes a wide range of disorders in animals and humans, affecting the gastrointestinal, reproductive, neuroendocrine, and immune systems [3–5].

The main cellular targets of DON are the ribosome and the ER [6, 7]. However, studies have indicated that DON-induced toxicity also induced oxidative stress and endocrine imbalance [8]. DON targets the mitochondria and causes the mitochondrial membrane potential (ΔΨ*m*) to decrease, leading to the deformation of the mitochondria and the subsequent release of cytochrome *c* into the cytoplasm [9–11]. Mitochondrial impairment occurred in the livers of fetuses when their mothers consumed DON [12]. Moreover, DON reduced intracellular hormone levels, including those of insulin, leptin, insulin-like growth factor 1 (IGF-1), and IGF acid-labile subunit (IGFALS), which could potentially cause DON-induced growth retardation [13, 14]. We recently discovered that DON inhibited the synthesis of growth hormone (Gh1) in rat GH3 cells, by reducing the cell viability and by inducing apoptosis [15]. Thus, we hypothesized that protecting cells from DON-induced cytotoxicity would prevent growth retardation.

Previous studies have identified that the NF-*κ*B signaling pathway, which occurs downstream of MAPK signaling, can be widely activated after DON treatment in the human Caco-2 and HT-29 cell lines [16, 17]. NF-*κ*B is activated by cytokines, such as TNF*α* and interleukin (IL), and regulates downstream effects on cell function [18, 19]. It regulates downstream antioxidant and prooxidant genes such as inducible nitric oxide synthase (iNOS), neuronal nitric oxide synthase, superoxide dismutase, catalase, heme oxygenase-1, xanthine oxidoreductase, NADPH : quinone oxidoreductase, and cyclooxygenase-2 [20]. We previously found that the T-2 toxin induced the transcription of *Nfkbil1* and *Nfrkb* in GH3 cells [15], suggesting that the NF-*κ*B signaling pathway was critical to mycotoxin-induced toxicity.

The rat GH3 cell line is a clonal strain of rat pituitary tumor that can synthesize and secrete prolactin and growth hormone. Trichothecenes induce considerable toxicity in endocrine GH3 cells by causing mitochondrial dysfunction, growth hormone synthesis inhibition, cell apoptosis, and inflammation [15, 21]. Therefore, we used an *in vitro* model of GH3 cells to study the effects of the NF-*κ*B inhibitor, pyrrolidine dithiocarbamate (PDTC), on DON-induced mitochondrial dysfunction and apoptosis. We discovered the mechanisms of DON-induced cytotoxicity in relation to nitric oxide (NO) generation, oxidant-antioxidant balance, and NF-*κ*B activation. The morphological changes in DON-treated cells were determined using flow cytometry and transmission electron microscopy (TEM). The effect of PDTC on DON-induced cytotoxicity was evaluated by TEM, with particular focus on phosphoryl-NF-*κ*B p65 nuclear localization, iNOS expression, and mitochondrial injury. Protection from apoptosis was monitored by flow cytometry.

## 2. Materials and Methods

### 2.1. Reagents and Chemicals

DON was obtained from Sigma-Aldrich (St. Louis, MO, USA). PDTC was obtained from Beyotime (Shanghai, P.R. China). Anti-iNOS (ab15323) and anti-actin (ab1801) antibodies were purchased from Abcam (Cambridge, MA, USA). Anti-phospho-I*κ*B*α* (Ser32/36; 5A5), anti-phosphoryl-NF-*κ*B p65 (Ser536; 93H1), and peroxidase-coupled goat anti-rabbit and mouse IgG (H + L) secondary antibodies were obtained from CST (Danvers, MA, USA). The apoptosis detection kit (Annexin V-FITC) was purchased from BestBio (Shanghai, China).

### 2.2. Cell Culture

The cells were cultivated as previously reported [15, 21]. Briefly, the GH3 cells from passages 5 to 15 were cultivated in high-glucose DMEM (HyClone Laboratories, Inc., Logan, Utah, USA) with 10% heat-inactivated fetal bovine serum (FBS; Gibco BRL, Gaithersburg, MD, USA) and 1% penicillin-streptomycin (HyClone Laboratories, Inc., Logan, Utah, USA) at 37°C, in the presence of 5% CO_2_. After 24 hours of incubation, the culture medium was changed to high-glucose DMEM, and the cells were incubated with or without the test reagents (DON and PDTC) for the indicated time intervals.

For cytotoxicity analysis, the cells were seeded in a 96-well plate (at a density of 1 × 10^4^ cells per well) and treated with 0, 300, 600, or 1200 mg/L of DON [15]. For quantitative real-time PCR, western blot, and chemoimmunological assays, GH3 cells were seeded in a six-well plate (at a density of 1 × 10^5^ cells per well) with 2 mL of medium. For TEM, the cells were seeded in a 75 cm^2^ flask (at a density of 5 × 10^5^ cells per flask) with 12 mL of medium. In some experiments, the cells were treated with inhibitors for 45 min to an hour, then exposed to DON for 12 hours. All experiments were performed in triplicate on at least three independent occasions.

### 2.3. Quantitative Real-Time PCR (qRT-PCR)

Total RNA was extracted using TRIzol (Invitrogen, Breda, The Netherlands) [15] and analyzed by quantitative real-time RT-PCR using the iNOS gene specific primers (S: 5′CCTCAGGCTTGGGTCTTGTTA3′; AS: 5′ATCCTGTGTTGTTGGGCTGG3′) as previously described. Fold changes in mRNA expression levels were calculated using the 2^−ΔΔCt^ method [11].

### 2.4. Western Blotting

Total protein was extracted, quantified, separated on a 12% SDS-PAGE gel, and transferred onto a polyvinylidene fluoride membrane (Millipore, Billerica, MA, USA) as previously described [15]. Membranes were incubated with anti-actin (ab1801), anti-phospho-NF-*κ*B p65 (Ser536; 93H1; diluted 1 : 1000), anti-phospho-I*κ*B*α* (Ser32/36; 5A5; diluted 1 : 1000), and anti-iNOS (ab15323; diluted 1 : 500) antibodies overnight at 4°C, according to the manufacturer's instructions.

### 2.5. Oxidative Stress Indices

The CAT activity, malondialdehyde (MDA), SOD, and glutathione peroxidase (GSH-Px) levels were assessed using commercial kits (Nanjing Jiancheng BioEngineering Institute Co. Ltd., Nanjing, China).

### 2.6. Chemoimmunology of Phosphoryl-NF-*κ*B p65

Immunofluorescence was used to determine phosphoryl-NF-*κ*B p65 localization. GH3 cells were fixed with paraformaldehyde (v/v, 1/25) at 37°C for 10 minutes. They were then permeabilized with cold acetone at −20°C for 3 minutes. After a PBS wash (0.1 mM, pH 7.4), cells were saturated with 3% BSA in PBS for 30 minutes, and incubated with the anti-phosphoryl-NF-*κ*B p65 antibody (diluted 1 : 100) at 4°C overnight. After another PBS wash (0.1 mM, pH 7.4), the cells were incubated with the secondary antibody for 30 minutes at room temperature. Coverslips were washed twice with PBS (0.1 mM, pH 7.4), incubated with the goat anti-mouse IgG antibody conjugated with Alexa Fluor 555 (Cell Signaling Technology, Danvers, MA, USA) for 30 minutes in the dark, incubated in 5 *μ*M DAPI staining solution (Invitrogen) for 5 minutes, and then washed in PBS. The fluorescence was monitored using an UltraVIEW VoX confocal system (PerkinElmer, Co., Norwalk, CT, USA).

### 2.7. GH3 Cell Morphology by Transmission Electron Microscopy (TEM)

The morphological variation in mitochondria was investigated as described earlier [21]. Briefly, the cells were fixed with glutaraldehyde (v/v, 2.5/100), postfixed in osmium tetroxide (w/v, 1/100), dehydrated in absolute ethanol, then embedded stepwise by polymerization at 45°C for 12 hours and at 60°C for 36–48 hours. The 70 nm ultrathin slices were stained with lead citrate for 10 minutes and with uranyl acetate for 30 minutes. Finally, they were washed thrice with ddH_2_O and dried. The slices were viewed with the H-7650 TEM (Hitachi, Japan).

### 2.8. Cell Apoptosis

The cells were harvested, washed, and centrifuged (2000 ×g, 4°C, 10 min). Then, they were suspended in Annexin V-FITC binding buffer at a density of 1 × 10^6^ cells per mL. They were then incubated with 10 *μ*L of propidium iodide (PI) solution (BD BioScience, San Jose, CA, USA) in the dark for 15 minutes. Apoptosis was measured using CyAn ADP as described previously [11].

### 2.9. Statistical Analyses

Data were analyzed by performing a two-way analysis of variance using the SPSS software (SPSS Inc., version 17.0, Chicago, IL, USA). *P* values < 0.05 indicated statistical significance.

## 3. Results and Discussion

Oxidative stress plays a major role in the mediation of cellular damage and dysfunction. It is inseparably linked to mitochondrial dysfunction and cell apoptosis [22, 23]. Free radicals contribute to the development of mycotoxicosis by inducing lipid peroxidation and changes in antioxidant status, and by causing the loss of cellular mitochondrial membrane potential [24, 25]. In this study, we found that after DON treatment, the activity of MDA and antioxidant enzymes such as CAT and SOD significantly increased, whereas the activity of GSH-Px significantly decreased (Table 1). This result corroborates other studies [26, 27] in which T-2 toxin exposure was associated with significant decreases in GSH-Px activity in granulosa cells from rats and in hepatic cells from chicken. However, a significant increase in GSH-Px activity was observed in DON-treated HT-29 cells [16]. Because GSH-Px functions as a scavenger of lipid peroxides and is induced by reactive oxygen species and hydroxyl free radicals in cells, the reduction in GSH-Px activity indicates a serious oxidant-antioxidant imbalance in cells. This implies that the rat GH3 cell line is likely more sensitive to DON toxicity than the human HT-29 cell line.

**Figure 1:**
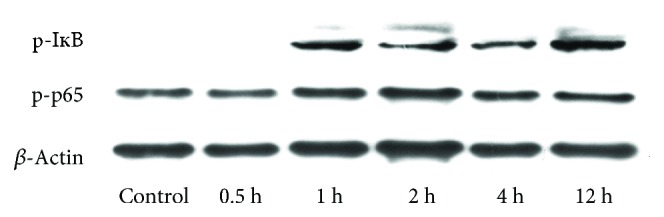
DON-induced I*κ*B*α* and NF-*κ*B p65 phosphorylation in GH3 cells.

**Figure 2:**
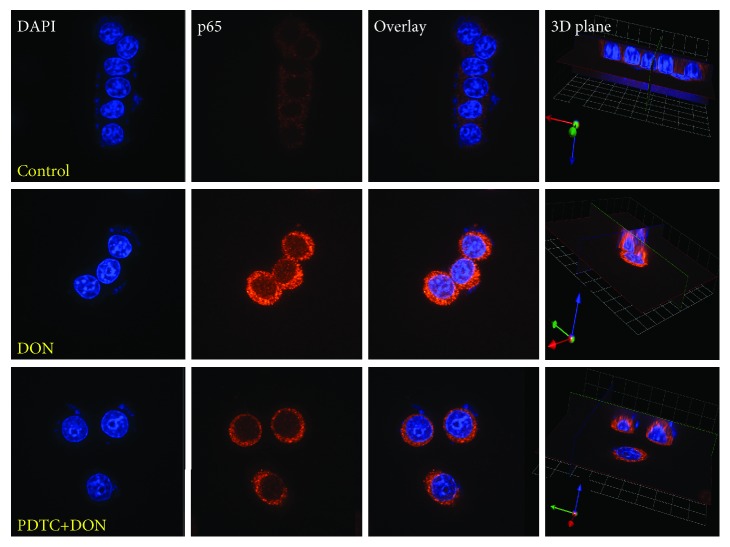
Nuclear translocation of phosphoryl-NF-*κ*B p65 (p-p65 (Ser536)) induced by DON treatment (1200 ng/mL) and PDTC pretreatment (20 *μ*M, 45 min) followed by DON treatment (1200 ng/mL) in GH3 cells, visualized through indirect immunofluorescence, using Alexa Fluor-conjugated secondary antibody. The nucleus was stained with PI. The panels show PI staining, Alexa Fluor staining, overlay, and the 3D plane of the cells. All photos were captured at 400x magnification. Phosphoryl-NF-*κ*B p65 was upregulated and can be observed in the nucleus.

**Figure 3:**
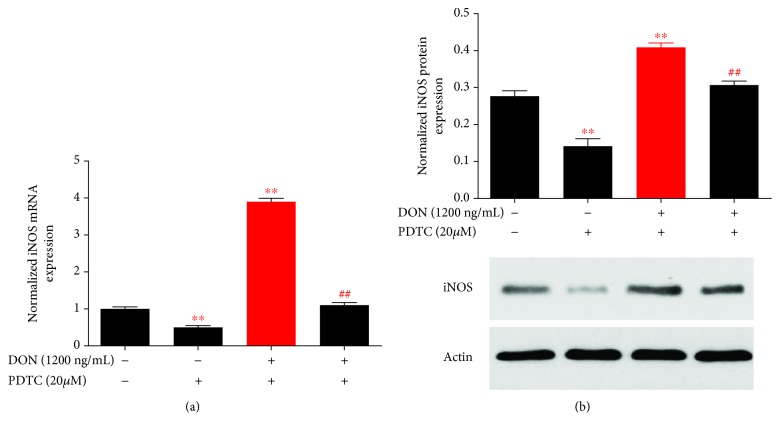
PDTC protects cells from DON-induced iNOS expression. (a) Cells were treated with DON, PDTC, and PDTC pretreatment followed by DON to assess iNOS transcription by qRT-PCR. (b). Cells were treated with DON, DON, PDTC, and PDTC pretreatment followed by DON to assess iNOS expression by western blotting. *P* values < 0.05 are indicated by a single asterisk, ^∗^. *P* values < 0.01 are indicated by double asterisks, ^∗∗^.

The NF-*κ*B transcription factors control many processes such as immunity, oxidative stress, and apoptosis. Phosphorylation of p65 NF-*κ*B at serine 536 is mediated by multiple protein kinases, including the I*κ*B kinase [28]. We found that DON induced the phosphorylation of I*κ*B*α* kinase as well as the phosphorylation and nuclear translocation of the p65 proteins (Figure 1). The pretreatment of cells with PDTC before DON treatment resulted in reduced p65 phosphorylation and translocation (Figure 2). In the nucleus, NF-*κ*B p65 binds to the iNOS gene promoter and upregulates its gene expression [29]. Therefore, we also investigated the iNOS mRNA and protein levels. The quantitative RT-PCR showed that *iNOS* gene expression increased after DON treatment, but decreased significantly with PDTC pretreatment (Figure 3(a)). The immunoblotting analysis showed similar patterns for the protein levels (Figure 3(b)). Reactive nitrogen species (RNS) and ROS are free radicals that cause oxidative stress. ROS generation did not significantly increase after DON treatment in human HT-29 cells [30] and RAW264.7 cells [31], whereas it increased significantly in HepG-2 cells [24]. At doses of 250 and 500 ng/mL, DON resulted in increased ROS and RNS production in human HT-29 cells [16]. Taken together, the PDTC appears to act as an antioxidant for DON-induced oxidative stress.

**Figure 4:**
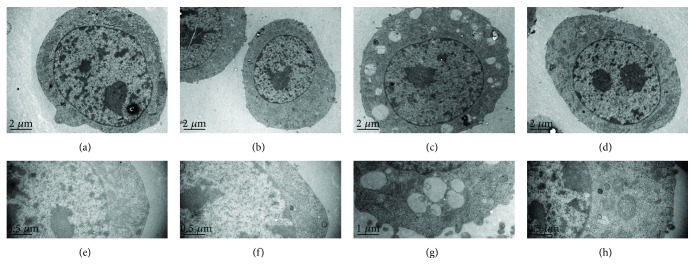
PDTC protects cells from DON-induced mitochondrial injury in GH3 cells. Cells were treated with DON, PDTC, and PDTC pretreatment, followed by treatment with DON for 12 hours. (a, e) Cell treated with DON showing normal mitochondria. (b, f) Cell treated with PDTC showing normal mitochondria. (c, g) Cells with PDTC pretreatment, followed by treatment with DON for 12 hours showing normal mitochondria and tiny vacuoles.

**Figure 5:**
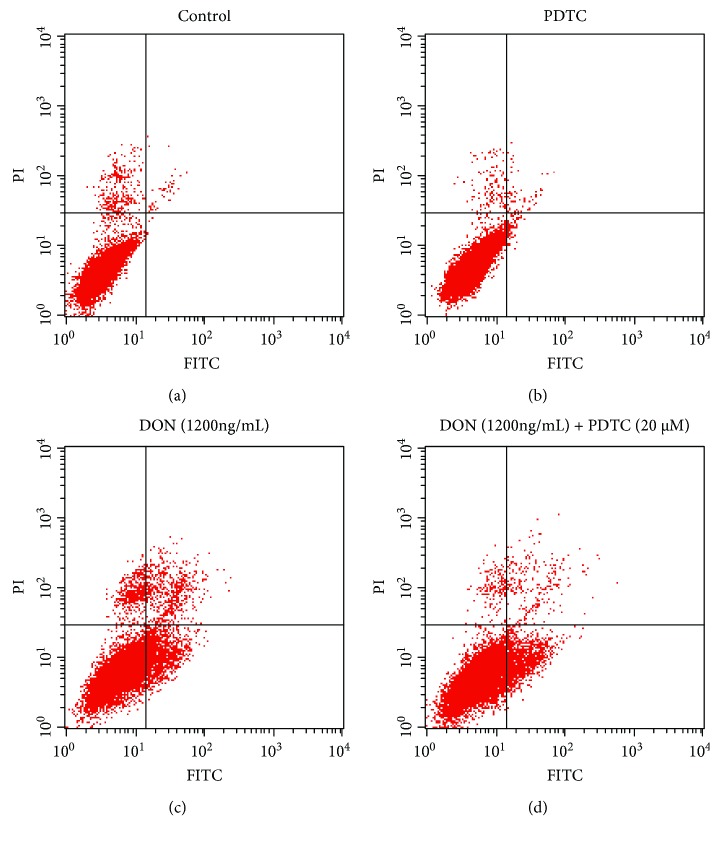
PDTC protects cells from DON-induced apoptosis in GH3 cells. Cells in the control group (a), cells treated with PDTC (b) and DON (c), and cells pretreated with PDTC followed by DON treatment (d) were used to assess the apoptosis rate. Data are shown as means for three separate experiments performed in triplicate.

**Figure 6:**
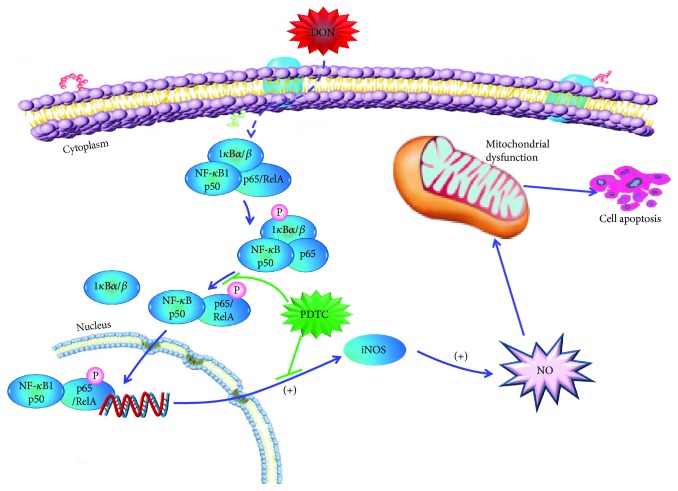
A proposed mechanism of action for the protective effect of PDTC in DON-mediated mitochondrial dysfunction and apoptosis. DON indirectly activates the NF-*κ*B signal pathway via the classical route of I*κ*B/NF-*κ*B p65 signaling. PDTC inhibits the translocation of NF-*κ*B p65 and the transcription of iNOS, and thereby protects cells from mitochondrial dysfunction and cell apoptosis.

In several cell lines, treatment with DON results in a loss of ΔΨ*m*, mitochondrial damage, caspase activation, and apoptosis [9–12, 16, 32]. PDTC relieves oxidative stress and improves mitochondrial structural integrity [33]. Hence, we tested the effects of PDTC pretreatment in DON-treated GH3 cells, with particular focus on mitochondrial ultrastructure and apoptosis. The control cells and PDTC-treated cells exhibited normal mitochondria (Figures 4(a), 4(b), 4(e), and 4(f)), whereas cells treated with DON for 12 hours displayed dose-dependent mitochondrial swelling, serious vacuolar degeneration, disarrayed cristae, and reduced electron density of the matrix (Figures 4(c) and 4(g)). PDTC reduced the DON-induced toxicity, and normal mitochondria were observed despite the reduction in vacuole size observed in the PDTC-pretreated cells (Figures 4(d) and 4(h)). DON treatment led to a significant increase in the number of early and late apoptotic cells. The proportion of apoptotic cells significantly decreased in DON-treated cells that were pretreated with PDTC (Figure 5). All of our findings suggest that PDTC inactivates NF-*κ*B, inhibits iNOS expression, and protects cells from cytotoxicity and mitochondrial toxicity via antioxidant effects (Figure 6). Our results are consistent with the known activity of another antioxidant, lutein, that protects cells from DON-induced mitochondrial structural damage, probably via inhibition of NF-*κ*B [16].

## 4. Conclusion

In DON-treated GH3 cells, DON caused the translocation of NF-*κ*B and induced iNOS expression. PDTC prevented the DON-induced migration of phosphoryl-NF-*κ*B p65 into the nucleus, inhibited DON-induced iNOS expression, and prevented DON-induced mitochondrial dysfunction and apoptosis.

## Figures and Tables

**Table 1:** Activities of GSH-Px, GST, CAT, and SOD in GH3 cells.

Group (ng/mL)	CAT activity (U/mg protein)	SOD (U/mL)	GSH-Px (U/mg protein)	MDA (*μ*mol/mg protein)
0	0.407 ± 0.008	1.907 ± 0.224	150.262 ± 0.001	1.476 ± 0.023
300	0.949 ± 0.012^∗^	2.938 ± 0.426^∗^	134.592 ± 0.034^∗^	1.736 ± 0.032^∗^
600	1.129 ± 0.006^∗^	4.630 ± 0.075^∗∗^	85.149 ± 0.217^∗∗^	1.698 ± 0.038^∗^
1200	2.326 ± 0.005^∗∗^	5.798 ± 0.257^∗∗^	53.719 ± 0.421^∗∗^	1.777 ± 0.042^∗^

*Note*: CAT 1 U = the amount of enzyme that consumes 1 nmole H_2_O_2_/min. GST 1 U = the amount of enzyme that conjugates 1 *μ*mole CDNB/min. GSH-Px 1 U = the amount of enzyme that converts 1 *μ*M GSH to GSSG in the presence of H_2_O_2_/min. SOD 1 U = the amount of enzyme required for 50% inhibition of pyrogallol autooxidation. Data are shown as means ± SD (*n* = 3) from three separate experiments performed in triplicate. ^∗^ indicates *P* values < 0.05, and ^∗∗^ indicates *P* values < 0.01.

## Data Availability

The data used to support the findings of this study are available from the corresponding author upon request.

## Abbreviations

DON: Deoxynivalenol
ERK: Extracellular signal-regulated kinase
FBS: Fetal bovine serum
FITC: Fluorescein isothiocyanate
GSH-Px: Glutathione peroxidase
Hck: Hematopoietic cell kinase
iNOS: Inducible nitric oxide synthase
LDH: Lactate dehydrogenase
L-NAME: L-NG-nitro arginine methyl ester
MDA: Malondialdehyde
NF-*κ*B: Nuclear factor-kappa B
PBS: Phosphate-buffered saline
PDTC: Pyrrolidine dithiocarbamate
PI: Propidium iodide
qRT-PCR: Quantitative real-time PCR
ROS: Reactive oxygen species
SMT: S-methyl-isothiourea
SOD: Superoxide dismutase
TEM: Transmission electron microscopy.
